# “We are not allowed to give any pain relief” — Swedish mountain-rescue operations from the perspective of mountain-rescue personnel: an interview study

**DOI:** 10.1186/s12873-026-01624-6

**Published:** 2026-05-27

**Authors:** Amanda Gezelius, Anton Westman, Johan Hylander

**Affiliations:** 1 Centre for Disaster Medicine, Department of Diagnostics and Intervention, Umeå University, Umeå, 901 87 Sweden; 2 Department of Anaesthesia and Intensive Care Medicine, Karolinska University Hospital, Huddinge, Stockholm, Sweden; 3 Department of Surgical Sciences, Anaesthesiology and Intensive Care Medicine, Uppsala University, Uppsala, Sweden

**Keywords:** Mountain rescue, Wilderness medicine, Major incident, Incident management, Disaster medicine

## Abstract

**Background:**

Rescue efforts in mountainous terrain remain a challenge for responding organizations such as police, emergency medical services and the mountain rescuers. This study aimed to shed light on Swedish mountain-rescue operations from the perspective of mountain rescuers.

**Methods:**

Individual interviews were conducted with 21 Swedish mountain rescuers from four different counties bordering the 1,700-km long and 300-km wide Swedish/Norwegian mountain range “The Scandes.” The collected data were analyzed using qualitative content analysis.

**Results:**

The overall theme was “A unique environment demands a tailored response.” Five categories were identified: (1) major incident preparedness, (2) intra-organizational preconditions, (3) initial incident response, (4) welfare concerns on-route and on-site, and (5) a difficult injury assessment.

**Conclusion:**

Intra-organizational bureaucracy may impede Swedish mountain-rescue response. Legislative issues regarding timely access to helicopters for the transportation of personnel and injured, as well as the restrictions on the administration of pain-relief drugs by licensed medical professionals, need to be addressed to decrease time to definitive treatment and improve patient comfort in future rescue efforts.

**Supplementary Information:**

The online version contains supplementary material available at 10.1186/s12873-026-01624-6.

## Background

 Mountainous areas serve as both residential locations and popular tourist destinations worldwide. The largest mountain range in Sweden, known as “The Scandes,” stretches 1,700-km long and 300-km wide, forming a natural border between Norway and Sweden [1]. The mountainous areas include deep valleys, rivers, lakes, marshlands, and forests that attract locals and tourists for various outdoor activities, including winter sports, mountaineering, hiking, canoeing, snowmobiling, and mountain biking [2]. In addition to the unique landscape, the weather in these areas may be unpredictable [3], which may offer challenges to the prehospital management, including response and overall mission time [4]. Rescuers may face both time critical conditions and a wide range of injuries and medical conditions that may require treatment during prolonged extractions [5–8].

The Swedish Mountain Rescue (in Swedish: Fjällräddare) is a volunteer organization comprising approximately 400 rescuers. These volunteers are organized into more than 30 rescue groups, each with a designated geographical area. Some rescue groups specialize in cave or high-altitude rescue. The organization is governed by the Swedish police, who hold overall operational responsibility. The police provide the volunteers with appropriate training and access to suitable equipment. Swedish mountain rescuers take a 1-week basic training course covering topics such as first-aid, avalanche and snow knowledge, radio communication, and navigation. Also, summer and winter exercises are conducted each year [9]. Instructors in first-aid courses are licensed medical professionals (e.g. physicians or nurses) who are active in the mountain rescue service themselves, which provides a tailored education for the mountain rescuers (personal communication Stefan Källström, May 13, 2026). During the period of 2018–2022, 771 non-fatal traumatic injuries occurred in the Swedish mountains [10]. Consequently, Swedish mountain rescuers need to be properly trained and equipped to treat severely injured persons. Each rescue group have an incident commander who receives a 4-day long education in incident management, similar to the education given to Swedish police commanders. This is deemed important as the rescue group incident commander collaborate with regional police commander and police officers and need to be accustomed to the nomenclature used during operations. The rescue group incident commanders usually solve day-to-day operations by themselves (up to 90% of rescue operations) (personal communication Stefan Källström, May 13, 2026).

How medical management is conducted in Swedish mountain rescue operations has not been studied. Gaining insight into the experiences of mountain rescuers could provide valuable information concerning everyday operations and identifying areas of improvement. Hence, this study aimed to explore the experiences of Swedish mountain rescuers participating in mountain-rescue operations. The research question was: How does Swedish mountain rescuers plan, execute and conclude a rescue effort from a medical and management standpoint?

## Methods

### Study design and context

This study used a qualitative design and consisted of individual interviews with Swedish mountain rescuers. The typical mountain rescuer should be between 18 and 65 years of age, own a suitable snow mobile and have a license to operate it, good geographic knowledge and be accepted by the local mountain rescue group [11]. Upon acceptation to the local mountain rescue group, the member uses their own snow mobile when going on rescue missions (with added insurance coverage from the police, should the snow mobile become damaged). The member also owns the right to access areas normal off limits to the public – even outside of the rescue missions to increase the members knowledge of the catchment area. During these reconnaissance expeditions, the member needs to carry a specialized terrestrial trunked radio unit and wear a mountain rescue uniform. Insurance via the police does not cover snow mobile damage during these reconnaissance expeditions. However, the member is insured (covering personal injuries) during missions, education and exercises. As the Swedish Mountain Rescue is govern by the police, the rescuers may also be tasked with search and rescue missions outside mountainous terrain upon reasonable request [11]. Still, the main responsibilities concerns aiding people in need, providing first-aid and transporting injured persons to the nearest accessible road and hand the injured over to the emergency medical services for further treatment and transport to a healthcare facility.

If an incident occurs in the Swedish mountains, those in need calls the national emergency number (i.e. 112), and are then transferred to the police regional command who in turn dispatches the closest mountain rescue group. The actual dispatch procedure is conducted through the *rapid reach system*, where dispatch information is sent out via a phone call (where operational details is read by an automated voice) and a text message, this double featured system is used to limit the possibility that information is lost, e.g. if the voice message becomes distorted due to bad cell coverage (personal communication Stefan Källström, May 13, 2026). A typical rescue operation described by a mountain rescuer is presented in Table 1, providing examples of the kinds of incidents mountain rescuers may encounter. A qualitative study design was selected to gain a better understanding of the experiences of mountain rescuers and their views on how Swedish mountain-rescue operations were conducted. Through interviews, participants may be encouraged to share in-depth descriptions of their experiences [12]. The interviews were analyzed using qualitative content analysis [13].

**Table 1:** Incident description from interviewee no 5

A frontal collision involving two snowmobiles occurred on one of the local snowmobile trails in a mountainous region in Sweden. Two mountain rescuers were dispatched. Upon arrival at the scene, one person was identified to have a serious head wound and suffering from amnesia. The second person involved was in pain but appeared to be stable. The person with a head wound was prioritized and transported to a hospital using a helicopter. The second person was transported by rescue sled down to the nearest accessible road to a waiting ambulance. The mountain rescuers were later informed that the second person had sustained complex pelvic fractures. The rescue operation included different organizations as for example, emergency dispatch, incident commanders from the police, mountain rescuers, emergency medical services and helicopters.	

### Participants and data collection

The inclusion criteria for participation were: current or previous employment in the Swedish Mountain Rescue, having participated in a mountain-rescue operation, and having provided informed consent to participate. The exclusion criteria were: no current or previous employment in the Swedish Mountain Rescue and lack of real experience in mountain-rescue operations (e.g., experience only from exercises).

### Recruitment

The police officer with national responsibility for the Swedish mountain rescue was contacted via email with a request to supply the researchers with contact information to mountain rescuers who might want to participate in a study concerning mountain rescue operations. Contact information (private or work email addresses) was received to twenty-one would-be participants from the entire mountain rescue services. JH sent an email to would-be participants containing written information related to the study and its aim. All twenty-one responded positively, see the discussion section regarding potential skewness.

Twenty-one (*n* = 21) interviews were conducted with mountain rescuers from all four counties which borders The Scandes, with a slightly uneven distribution of participants from the different counties (Table 2). The participants had different backgrounds, some with no healthcare education, some with extended first-aid courses (for example as ski patrollers, services members in the armed forces or rescue services personnel) and some with dedicated education as for example registered nurses with 1-year of sub-specialization working in emergency medical services. Their experience of working in the Swedish Mountain Rescue ranged from 6 to 41 years.

The interviews were conducted using an internet-based application for video meetings (Microsoft Teams), which allowed the interviewers and interviewees to see and hear each other. One or two interviewers conducted each interview using a semi-structured interview guide (modified from Hylander et al. [14]) consisting of open-ended questions to allow the participants to answer with more vivid answers than just yes or no (Appendix 1). This also encouraged participants to identify and express possible challenges themselves [12]. The interview guide remained unchanged throughout the study period. Interviews were conducted and recorded digitally from January to June 2024. The collected data was stored on a cloud based secure server owned by Umeå university.

**Table 2:** Description of participants

Years in mountain rescue	Health care background or extended first-aid training	County
6	Ski patroller	Norrbotten
32	None	Dalarna
18	Ambulance nurse	Jämtland
41	None	Norrbotten
8	Active services in the armed forces	Västerbotten
22	Rescue services	Västerbotten
6	Rescue services	Jämtland
17	Rescue services	Västerbotten
5	None	Jämtland
13	None	Västerbotten
25	Ski patroller	Dalarna
7	Anesthesia nurse, ski patroller	Jämtland
25	None	Norrbotten
6	None	Västerbotten
39	None	Norrbotten
5	None	Jämtland
23	None	Västerbotten
35	None	Norrbotten
10	None	Västerbotten
25	Anesthesia nurse, rescue services	Norrbotten
25	Ambulance nurse, rescue services	Norrbotten

### Data analysis

The recorded interviews (total duration 18 h 51 min 32 s) were transcribed verbatim (in total 291 pages and 165 226 words) by AG and a research assistant. Prior to the coding phase, each interview was read by the authors to gain an idea of the content of the interview. Coding was then performed using an inductive method in which codes were formed from the data during the analysis process [15]. Examples of the process of forming codes are presented in Table 3. Latent (what was said between the lines) and manifest (what was actually said) coding were used in this study. The codes were sorted into different subcategories and further into categories [13]. During the process, the data was analyzed back and forth, re-forming codes if needed to ensure that the coding matched the interview’s context. A theme, or “red thread”, which binds the categories together were also identified. The data, coding, and categories and theme were discussed among the authors until a consensus was reached.

**Table 3:** Examples of meaning units, condensed meaning units, codes, subcategories and categories

Meaning unit	Condensed meaning unit	Code	Subcategory	Category
Well, if I can dream, I would ask for a pain relief medication that I could administer. Maybe an inhalator of some sort. This would help to take the edge off the pain and provide some anxiety relief.	Asking for an inhalator medication for pain and anxiety relief.	Request of pain relief to give to patients	Achieving balance in equipment requirements	A difficult injury assessment
In this case, it worked out fine. This since all parties involved, including the ambulance driver, were familiar with the area. We were able to agree on a meeting point because everyone knew where a particular person lived. Our exact words were something like “We can meet 500 meter before reaching XX’s house”. This approach doesn’t always work.	Involved parties were all familiar with the area, and this made it possible to easily decide on a meet-up position.	Local knowledge is an advantage for identifying position	The importance of personal qualities	Intra-organizational preconditions

### Ethical considerations

The study was conducted in accordance with the principles of the Declaration of Helsinki [16]. Recorded informed consent was obtained from all participants. Participants were given pseudonyms (e.g., interviewee 9), ensuring that quotes from the interviews could not be traced back to any specific mountain rescuer. No economic compensation was provided for participation. The study received prior approval from the Swedish Ethical Review Authority (Dnr: 2023-06563-01).

## Results

One overarching theme and five main categories were identified during the analysis. Table 4 presents each category and associated subcategories. Quotes are used throughout the Results section to demonstrate transparency.

**Table 4:** Theme, categories, and subcategories

Theme	Category	Subcategory
A unique environment demands a tailored response	Major incident preparedness	Cooperation as central element Confidence in handling a mass casualty event Uncertain personnel availability Effective communication is vital
	Intra-organizational preconditions	The importance of personal qualities Unavoidable geographical demands Access to training and educational resources
	Initial incident response	Decentralized Emergency call operators Remote coordination by police personnel Specific demand for helicopter assistance
	Welfare concerns on-route and on-site	Planning for potential hazards Ongoing risk assessment Active risk mitigation Risk acceptance
	A difficult injury assessment	Limitations in medical knowledge Achieving balance in equipment Warranted feedback

### Theme: A unique environment demands a tailored response

During the analysis, one theme emerged, “A unique environment demands a tailored response.” The uniqueness of the mountains presents various challenges, including transport, communication, coordination, cooperation, hazard management, and dealing with unpredictable weather conditions, which may aggravate preexisting medical conditions, impact assessment and access to an incident site. Moreover, the environment reflects the qualities that a Swedish mountain rescuer should possess to cope with a demanding and changing environment.

#### Category 1: Major incident preparedness

The ability to effectively communicate and collaborate with other organizations is considered an important factor in Swedish mountain-rescue operations, particularly during major incidents. Mountain rescuers remain confident in their capacity to manage such incidents, while also recognizing the accompanying challenges.

##### Subcategory: Cooperation as central element

Many mountain rescuers have emphasized that cooperative skills were fundamental to rescue operations in mountainous regions, especially during potential mass-casualty events. Rescue operations involved coordination with various entities, including the police, ambulance services, rescue services, Swedish armed forces, and Norwegian helicopter services. Overall, cooperation and communication between organizations were described as effective. However, challenges arose in larger rescue operations where numerous personnel were involved, leading to unclear roles and an added risk of tasks being overlooked. This complexity was mentioned as being particularly difficult to navigate at the management level.

It was difficult to obtain clearly defined responsibilities and to know whom to follow. /…/ A structure eventually emerged there as well, but it took time before roles and lines of authority were clearly established.

Interviewee 15

##### Subcategory: Confidence in handling a mass casualty incident

In the event of a mass-casualty incident, many mountain rescuers expressed confidence in the capability of the mountain-rescue organization to manage such situations. There was a strong belief in their ability to swiftly mobilize a significant number of personnel, including the swift mobilization of additional mountain-rescue groups from nearby regions. However, concerns were raised regarding the number of casualties that a single rescue team could handle without lowering standards of care. They estimated their maximum capacity was to care for approximately 2–4 individuals simultaneously. The mountain rescuers acknowledged that the exact number was difficult to determine, because each rescue operation was unique and dependent on the available personnel and other variables, such as the degree of injury and access to the injured individual.

Well… we kind of already have, since we’re spread out, we have good coverage across the area where we operate, so in many cases we can be on site quickly.

Interviewee 14

##### Subcategory: Uncertain personnel availability

Interviewees described the uncertainty of available personnel could impact the organizations’ ability to handle major incidents. Given that mountain-rescue organizations are volunteer-based, there is a risk of a limited number of responders or, in worse cases, the risk that none would respond to the call. No on-call functions have been built into the system. Additionally, those who responded may be far away and lack access to their equipment, which could result in delays and in increased response times. Another challenge was the recruitment of new members to the mountain-rescue teams, as the rural areas are scarcely populated, limiting the number of potential candidates.

Yeah, I mean, we’re talking about really small communities when it comes to rescue operations. There aren’t that many permanent residents in the Swedish mountain range — maybe 10 to 12 people — and even then, you need someone who’s the right age, someone who can drive a snowmobile. You can’t be too young, because first of all, you need to be able to drive a snowmobile, and for the police, you can’t be too old either — the age limit is 65, then you have to stop. So that really limits the number of possible candidates.

Interviewee 10

##### Subcategory: Effective communication is vital

Communication was highlighted by the participants as a key factor for a successful rescue mission. In the event of a major incident, the demands for effective communication increased. Major incident rescue operations and communication between different agencies were described as working well, although it could be challenging to maintain effective communication throughout prolonged incidents. To combat limited cell phone coverage participants described that they also used terrestrial trunked radios provided by the police and donated *in-reach units* (where emergency communication is conducted using short text messages). Another challenge mentioned in larger rescue operations was the risk of tasks falling through the cracks when there was an unclear distribution of tasks and/or responsibilities within and between multiple organizations.

Deciding who should talk to whom and how everything should be organized is challenging. There’s an ideal model, but reality rarely matches it, so we adapt and do the best we can.

Interviewee 17

#### Category 2: Intra-organizational preconditions

Swedish mountain-rescue organizations must adapt their operations to unavoidable factors, such as vast terrain and the unique challenges of working in an environment where specific qualities may offer advantages.

##### Subcategory: the importance of personal qualities

Respondents stated that the organization depended on the qualities of individual mountain rescuers, such as having a personal network of contacts, good local knowledge, their own equipment, and a high level of ingenuity. For example, planning or solving unexpected events, such as quickly finding a replacement snowmobile or calling a friend for specific weather and terrain updates for an operation in a remote area. Many mountain rescuers have also emphasized the importance of local knowledge in the unique setting of mountainous areas, because understanding the area is crucial for planning and solving challenges in unfamiliar terrain.

It is based on personal commitment, or this would never work, the mountain rescue effort. This is not working because of the police providing us with what we need, this is working based on our commitment to make it work.

Interviewee 9

##### Subcategory: Unavoidable geographical demands

The mountainous area itself was described as a challenging factor by multiple mountain rescuers. The respondents stated that mountainous terrain limits the conditions for rescue operations because it may be difficult to navigate. Additionally, weather and distance prolonged the time required to reach the incident site. Blizzards, wind, and cold were considered challenging conditions also limited the possibility of using helicopters for transportation. Other geographical aspects associated with the Swedish mountains included navigation through a landscape of rivers, thin ice, glacial crevasses, and avalanche-prone terrain. The environment was also described as impairing the ability to communicate, especially in areas with limited reception, which was partly due to mountains impeding radio communication and the overall lack of radio towers to cover vast areas.

Since there are no radio towers up here, our signal coverage disappears after one kilometer out on the mountains.

Interviewee 1

##### Subcategory: Access to training and educational resources

Regarding training and education, the opinions and individual needs of mountain rescuers varied. Some felt that their current training levels were sufficient, whereas others advocated for more extensive training. Most participants agreed that the quality of the training was good. However, different mountain-rescue groups reported varying number of training hours and approaches. Training together with other mountain-rescue personnel was emphasized as important for learning, building camaraderie, and enhancing teamwork.

People simply don’t have the time; they have many other commitments, which makes it difficult for volunteer organizations to function. In the end, participation is voluntary.

Interviewee 7

#### Category 3: Initial incident response

Mountain rescuers described the initial dispatch procedure as a series of separate events. The process included emergency call operators, the police regional command center, and an overall police incident commander. The interviewees stated that this chain of events could be time-consuming, potentially delaying their dispatch to the incident site. Many interviewees also raised concerns about their communication with the regional command centers.

##### Subcategory: Decentralized Emergency call operators

Several mountain rescuers described the emergency call system as time consuming because of the many stages of decision-making involved, with the first stage being the emergency call operators receiving an initial call. From the perspective of the mountain rescuers, it was believed to be challenging for the emergency call operators to determine whether an emergency call should be directed to mountain rescue, as it falls under a different jurisdiction than usual emergency services. Before alerting the mountain-rescue team, the emergency call operators needed to identify whether an emergency had occurred in a mountainous area. The emergency call operators may be located at various sites in Sweden, with limited knowledge of where the mountainous areas are located. This was described as a limiting and time-consuming step. One mountain rescuer proposed implementing a map filter in which mountainous areas were outlined to facilitate timely dispatch.

Everything takes time. If you have broken your foot or leg on the mountain and call the emergency number [112 – authors remark], then the emergency call operators need to realize they can’t send for an ambulance and then transfer it to the police. Then, the police must assess whether this is dangerous or not, can they fix it themselves? Can they send for a helicopter? This whole chain takes quite a long time before they decide that I as a mountain rescuer should even get dressed.

Interviewee 10

##### Subcategory: Remote coordination by police personnel

Mountain rescuers reported several challenges when working with and under the police regional command center. One of the challenges identified was that regional command center operators seemed to lack knowledge and understanding of what mountain rescuers do and what their missions demand. Some mountain rescuers expressed concerns about regional command center being located far from the mountain range. For example, one rescuer described a situation in which the regional command center operator made decisions based on their local weather conditions, which were irrelevant to rescuers who were several miles away at a different site with entirely different weather conditions. It was also reported that regional command center operators in some cases were changed during active rescue operations, which led to miscommunication and information being lost, further complicating the rescue effort.

I’ve been the incident commander for the past few years and have mostly been in contact with the regional command center during rescue operations. They’re incredibly friendly and cooperative. However, they lack understanding of where we are in the country, and that can cause some issues. For them, it often sounds like, ‘Oh, you guys just go handle this.’ Meanwhile, we know it will take us four hours to get there for something like a femur fracture, and we have to transport the patient.

Interviewee 20

##### Subcategory: Specific demand for helicopter assistance

In the Swedish mountains, the vastness of the different areas was described as a unique challenge for rescue operations. Interviewees pointed out regional differences when needing helicopter assistance compared with regular rescue services. Long distances often justified the use of helicopters, especially in emergencies where the need was even greater. However, some mountain rescuers noted that obtaining mandatory approval by the police commander for helicopter use could take a considerable amount of time, as police helicopters were the first choice before civilian helicopters owing to legislative reasons. To use a civilian helicopter (a helicopter that is used entirely for civilian purposes, in this example to transport tourists and hikers and as such does not carry stretchers, medical equipment or is staffed with health care professionals), a procurement agreement needed to be established between the Swedish police and the helicopter company. In a time-pressed setting, this restricts the use of civilian helicopters even if they are the closest available transport options. Many interviewees expressed frustration with inconsistent permissions regarding the use of civilian helicopters. One mountain rescuer recounted a situation in which a patient suffered a medical complication, compartment syndrome, owing to a prolonged waiting time. The rescuer explained that there was a civilian helicopter base closer to the incident scene, but they had to wait for a helicopter that was farther away.

Sometimes when we are on a rescue mission and request an ambulance helicopter, they cannot fly due to bad weather. Then it’s frustrating to get a negative response because of procurement agreements, when we request to use a private helicopter service nearby instead, who knows the area and can fly in such conditions.

Interviewee 8

#### Category 4: Welfare concerns on-route and on-site

Mountain rescuers stated that safety was an important aspect of their missions during mountain-rescue operations. This included multiple stages such as planning for probable hazards, continuous risk assessment throughout the operation, identification of ways to mitigate risks, and acceptance of a certain level of risk.

##### Subcategory: Planning for potential hazards

When an emergency call was received, several mountain rescuers explained that they immediately began planning the operation. This planning included deciding on which equipment to bring, the estimation of mission duration, the arrangement of proper transportation, and the identification of obvious and potential risks. One respondent stated that the uniqueness of each rescue operation made it difficult to anticipate every possible risk or unexpected situation. Although the use of general checklists for preparation was suggested, there were also concerns about the effectiveness of the checklists because rescue operations may vary greatly. Furthermore, in some scenarios (e.g., missing persons), it may be necessary to plan for 1–2 days of continuous operation, which added another layer of uncertainty.

You need to have some sort of backup plan, because things can go wrong. You might get stuck with the snowmobile or something. So, for me, it’s really about the situation, location, and response time — that’s how my mind works. Instead of getting stuck in the details of how we’re going to do this or that, I focus on staying a few steps ahead, as much as possible.

Interviewee 5

##### Subcategory: Ongoing risk assessment

To identify potential risks that were not covered during the planning stage of mountain-rescue operations, interviewees reported that they continuously conducted risk assessments. Part of this process includes group discussions, evaluating environmental conditions (e.g., changes in weather), and adjusting plans based on the evolving situation. Respondents identified several potential risks, including cold weather, avalanche risk, unsafe ice conditions (such as thin or cracked), navigating snowmobiles in otherwise inaccessible terrain, heavy snowfall, and high-stress situations. Some mountain-rescue groups, particularly those specializing in cave or high-altitude rescue, were reported to use predetermined checklists to consider various risks.

When we’re on a mission, it’s always ‘stop and go.’ – like can we keep going? How far can we go? Can we make it? Will we be able to get back on our own? Stop… that’s what we’re thinking about all the time. So, we’re constantly planning.

Interviewee 7

##### Subcategory: Active risk mitigation

Respondents acknowledged that they worked in a risk-filled environment. However, they also actively sought to minimize the identified risks. Many mountain-rescue groups had established local rules to mitigate certain risks elements and required at least four people to participate in a rescue operation. Other strategies for mitigating risks included using avalanche transceivers and ensuring that all equipment was tested before heading out. In some cases, if the risks to their own lives was deemed to be too high (e.g., extreme temperatures or high winds), rescuers may decide to decline the mission. Some interviewees stated that their respectively mountain-rescue groups worked to establish specific safety zones at the incident site (access was only provided if the personnel wore adequate protective equipment, such as helmets), a measure implemented to ensure that risks were minimized during rescue operations.

The ground rule is that we should be at least four people [during a mission -authors remark] We can be more, but we shouldn’t be fewer than four. For our own safety, to be able to help each other.

Interviewee 11

##### Subcategory: Risk acceptance

Because the processes of risk assessment and risk mitigation were ongoing throughout the rescue mission, some elements and aspects of the risks were accepted. The environmental risks include cold and snowy weather conditions. Some mountain rescuers indicated that they were not trained in rescue operations on open water. However, if someone were in need, it could be difficult for them to refrain from intervening. One rescuer acknowledged that while they would not be protected by insurance in such situations, they would choose to act anyway. This sentiment also extended to other scenarios, particularly life-or-death situations. For example, if the minimum number of participating mountain rescuers was not met (as mentioned above, some mountain-rescue groups required a minimum of four available rescuers before leaving the station), they could decide to proceed with the mission.

One mountain rescuer described the occurrence of work-related injuries (i.e., frostbite) despite trying to mitigate the identified risk assessment.

I’d also take my boat, even though I’m not expected to. We’re not trained to act in operations on water, but as I said, we’re action-oriented people. And… I’d do the same thing as my colleagues any day of the week. If I know there’s someone out in the lake and I have a boat that I regularly use on that lake, why wouldn’t I go out?

Interviewee 14

#### Category 5: A difficult injury assessment

This category emphasized the challenges of making on-scene assessments in emergency medical situations, particularly owing to varying levels of experience among mountain- rescue volunteers and potential limitations in equipment. Feedback on the injury outcome was desired so that the participants cold learn whether they could have done anything differently to improve the results.

##### Subcategory: Limitations in medical knowledge

The interviewees pointed out that the medical knowledge within the mountain-rescue teams varied significantly. For instance, some members work in healthcare, others are involved in mountain tourism, and others manage local stores. This diversity of knowledge was described as beneficial in some contexts, however, in cases involving severely injured persons, mountain rescuers may lack specialized healthcare training and need to rely on basic first-aid knowledge. This was descibed as a challenge particularly when treating people with medical emergencies. Many mountain rescuers emphasized that their primary mission was to stabilize patients and address the most urgent issues onsite and leaving the more complex medical care to professional healthcare providers.

We understood that there had been quite a bit of force involved. We understood how the accident happened, and from that, we could estimate how much energy was involved. It was clearly a lot of force involved and of course, she was in pain. But it’s difficult to assess how much damage there is and how much shock is involved in a situation like that.

Interviewee 8

##### Subcategory: Achieving balance in equipment

Most mountain rescuers who were interviewed expressed a general satisfaction with their equipment. It was described as important to maintain a balance between equipment requirements and limitations, such as packing space, total weight, and available resources. Maintaining balance was seen as useful for making the most effective use of the available equipment while still adhering to the goals of the specific mountain-rescue effort. Seven rescuers specifically requested pain-relief drugs, some stating that the availability of these drugs would improve the quality of care provided and facilitate the transportation of injured persons. The challenges in providing adequate pain relief to injured individuals were also raised. For example, administration and calculation of an appropriate dosage may require specific training. A specific concerns of administering pain relief drugs in mountain rescues was that licensed healthcare providers such as nurses and physicians were unable to bring or administer pain-relief medication when working as mountain rescuers. The reason for this was that different legislations governs healthcare and mountain-rescue personnel. Given that mountain rescuers are goverened by the police, they (independent of their healthcare background) do not fall under the Health and Medical Service Act that governs healthcare personnel. Consequently, injured individuals do not receive any pain-relief mediciation during mountain rescue operations.

We are not allowed to use any drugs at all, there is no chance. But we try. We have talked about an inhalator pain relief, which they use in other countries, like Australia and Canada. But nothing in Sweden, since it’s not approved by the National Board of Health and Welfare, we are not allowed to give any pain relief.

Interviewee 7

##### Subcategory: Warranted feedback

Upon the completion of a rescue operation, the usual practice was to document the event, which was usually undertaken by the team leader. Debriefing sessions were available if necessary. However, the interviewees expressed concerns about the lack of feedback regarding their written reports and patient outcomes, which was viewed as a missed opportunity for improvement. Another challenge mentioned was the psychological impact of working in familiar areas where the victim may be someone they knew. In these situations, having a strong support system within the group, along with the opportunity to participate in debriefing sessions, was described as important.

It would have been incredibly valuable to receive feedback. We have first-aid training, where we should be able to identify strokes and recognize diabetes. We learn about different types of illnesses and various cases. But when we assess something like a femur fracture, we never get any feedback from healthcare to know if we made the right decision regarding the care.

Interviewee 11

## Discussion

The main findings of this study were that legislative issues seem to hinder both the utilization of civilian helicopters to transport people or medical equipment and restricts licensed healthcare professionals from carrying or administering pain-relief medication to injured persons, if they are working as mountain rescuers. Both these aspects may impact the quality of care provided to those individuals injured in mountainous settings in Sweden.

In mountaineering, fractures to the extremities are the most common injuries observed in alpine trauma [17] approximately 10% may sustain critical multisystem injuries [18]. Other traumatic injuries may include snowmobile incidents and avalanches. Buried avalanche victims survival decline by 2–3% for each minute a victim is buried [19, 20] which means that mountain rescue may not even reach the incident site in time. For example, a Japanese study examining fatalities during mountain activities reported a survival rate of only 3.5% upon rescue arrival [8] suggesting that first aid training and buddy-rescue may be important. Education in first aid and buddy-rescue to hikers, for example by the Swedish Tourist Association, may be one way to limit time to treatment. It is essential to have the right knowledge and experience to provide appropriate care [21].

Given the remoteness of the Swedish mountain range, the time from dispatch to arrival of mountain rescuers at the scene may be considerable. It is worth noting that emergency dispatch centers receive approximately 3 million emergency calls per year, of which 300 calls (0.0001%) involve mountain rescues [22]. The multiple-step dispatch process could benefit of becoming more streamlined and coordinated. For example, by using the most appropriate means of transport. If the police helicopters are occupied, could other airborne resources from the armed forces, search and rescue or neighboring countries be used instead? Tomazin et al. argued that utilizing helicopter emergency medical services in mountain rescue can significantly reduce prehospital time [23]. However, the Swedish procurement model restricts the use of civilian helicopters (i.e. not police, military, search and rescue or medical) for Swedish mountain rescues. On the other hand, civilian helicopters may lack the necessary medical equipment (e.g., medical oxygen, first-aid kits, defibrillators, etcetera) or equipment to secure e.g., stretchers or multiple injured persons. In what manner different helicopters services could aid the Swedish police is under ongoing governmental inquiry and have yet to be determined [24].

Regarding health care, the Swedish Health and Medical Service Act govern the organization and provision of health and medical care at the national level. One of the central goals stated in The Swedish Health and Medical Service Act is “equal care for the entire population” [25]. When licensed healthcare personnel (who usually fall under the Health and Medical Service Act) volunteer as mountain rescuers, they are prohibited from administering pain-relief medication as they are then governed by the police and thus falls under the Law of Civil Protection, which does not address healthcare and medical aspects [26]. These legislative issues need to be addressed to improve the quality of care for mountain-rescue victims. A possible solution could be that mountain rescuers may administer pain-relief medication mandated by municipality physicians and local medical center as highlighted by others [27]. Some kind of senior medical advisors employed or linked to the mountain rescue service could also be of valuable in giving mountain rescuers feedback on injury outcome which in turn could facilitate learning and incite interest in medical management among the mountain rescuers.

### Limitations

Trustworthiness (including credibility, dependability, transferability) is an important aspect in qualitative content analysis [13]. Concerning *credibility* - how well the categories and themes cover the collected data - a description and example of how coding was carried out was given so readers may follow the process. Each of the subcategories, categories and theme were discussed among co-researchers until consensus was reached and quotes from the interviewees have been included to show consistency with the reported data. About *dependability* and if findings change over time, this might be the case if the interview questions changed during the data collection period. A strength in this aspect is that the interview guide was unaltered during the study period. However, there is a possibility that questions may be asked in a different way by each interviewer, this have been mitigated by using an open dialogue within the research group.

About *reflexivity* researchers need to be conscious of their own subjective biases, the experiences of the authors as healthcare providers may have introduced subconscious biases into the coding process. To counteract this, the initial read-through of the material and initial analysis was conducted separately, and different codes and categories were discussed among the authors until a consensus was reached. None of the of the authors had prior experience with mountain-rescue operations and thus did not have a prior understanding of the real-life experiences of mountain rescuers. This lack of experience may have led the respondents to be hesitant to share their experiences in a similar manner as sharing these experiences with a colleague. However, the answers provided by the respondents were vivid and rich in content which is important when considering *transferability*. Given the sparingly body of knowledge concerning Swedish mountain rescue services – detailed information of for example dispatch procedures and educational level of dispatchers is lacking. This may hamper the aspects of transferability as the context may seem vague. However, to the best of our knowledge, we have tried to describe the mountain rescue service in Sweden so other researcher may assess if the results are transferable to similar settings in other mountainous environments in for example Europe.

*Skewness and data saturation*. Element of skewness may have been introduced into the selection process. As contact information to would-be participants was given by the person responsible for the Swedish mountain rescue service, highly motivated individuals may have been selected. This could have been the case as all would-be participants chose to participate. We sought to include all that expressed a will to participate and gave their informed consent. Notable, there were an interesting distribution of different mountain rescuers with a slight uneven distribution. Hence, potential skewness was not limited to a certain district. As no compensation was given, financial motivation seems unlikely. We also found that the descriptions from the participants began to sound somewhat similar – indicating that data saturation had occurred. Yet a lot of individual experiences was reported by participants.

## Conclusions

Mountain rescuers experienced interorganizational challenges concerning dispatching procedures and lack of coordination. Legislative issues concerning timely access to helicopters for transportation, restrictions concerning administration of pain-relief drugs by licensed medical professionals and feedback regarding injury outcome need to be addressed to facilitate the time to definitive treatment. These suggestions may improve patient comfort and facilitate learning from incidents in this rugged setting.

## Electronic Supplementary Material

Below is the link to the electronic supplementary material.

Supplementary Material 1

## Data Availability

Dataset (in Swedish) is available from the corresponding author upon reasonable request.
