# Supplementary material for: “We are not allowed to give any pain relief” — Swedish mountain-rescue operations from the perspective of mountain-rescue personnel: an interview study
Source: BMC Emerg Med. 2026 May 27;26:156. doi: 10.1186/s12873-026-01624-6 (PMC13214282; doi:10.1186/s12873-026-01624-6)
Supplement: Supplementary file 1 — Supplementary Material 1 [file 12873_2026_1624_MOESM1_ESM.docx]

**Information to research participants: Mountain rescuers experiences of participating in mountain rescue operations in Swedish mountainous settings** *-Preliminary interview guide*

**Introduction**: Oral verification of the participant information sheet’s content. Introductory questions regarding years of service, level of education, and practical/theoretical experience in participating in rescue operations in Swedish mountainous environments.

**Scenario**: You have experience from one or more rescue operations in a mountainous environment. During these events, you acted in your professional role. Today's interview will focus on these events. Alternatively, is there a specific event that you remember particularly well?

**Questions:**Pre-operative phase
-What were your thoughts concerning the rescue effort when you received the call
-Please describe how you planned the work while en route to the scene.

Operative phase
*Command and control*
-Please describe how you experienced the collaboration from a command perspective.
- Would you like to describe your experiences of the operational work?
- How did your and other organizations collaborate at the command/dispatch center level?

*Safety*
- What does "safety" mean to you during an operation in a mountain environment?
- What are your thoughts on safety before a rescue operations in mountainous terrain? (e.g., describing conditions such as darkness or cold).
-Identification of risk factors? (e.g., planned access routes, risk of landslides/avalanches, etc.).

*Communication*
- How was the communication with the involved stakeholders/actors?
-How were arising problems solved? (e.g., taking initiative)

*The incident site*
-Can you tell us about your work at the scene of the incident?
-Who within your organization conducted the initial assessment of the incident area? What is included in this? How did you perceive that the initial assessment was communicated/coordinated with other organizations? Please provide examples of situations.

Post-operative phase
-Can you describe your thoughts after the operation? Any take home messages? Any difficulties?
-How did you review/debrief the operation afterward?
-How did you experience the collaboration with other actors after the operation?
-Did any routines changed? Did you miss any tools/equipment? Any lessons learned?
-How do you view your organization’s capability to handle a mass casualty incident in this environment? (e.g., staffing, identification of injuries, sorting/triage, treatment, and transport of victims).
